# Ultrasound-Assisted Liposuction for Idiopathic Buffalo Hump: Anatomical Considerations and Technical Implications

**DOI:** 10.3390/jcm15072704

**Published:** 2026-04-02

**Authors:** Jeeyoon Kim, Yerin Kim, Eunyoung Rha, Jongweon Shin

**Affiliations:** Department of Plastic and Reconstructive Surgery, Eunpyeong St. Mary’s Hospital, College of Medicine, The Catholic University of Korea, Tongil-ro 1021, Seoul 03312, Republic of Korea; jeeyk017@catholic.ac.kr (J.K.); hollyn6710@naver.com (Y.K.); reyrha@catholic.ac.kr (E.R.)

**Keywords:** posterior neck lipomatosis, buffalo hump, ultrasound-assisted liposuction, dorsocervical anatomy, fibrofatty tissue

## Abstract

**Background:** Posterior neck lipomatosis, commonly referred to as a buffalo hump, is characterized by abnormal accumulation of adipose and fibrofatty tissue in the dorsocervical region, leading to aesthetic deformity and functional discomfort. Ultrasound-assisted liposuction (UAL) has been widely used as a minimally invasive treatment option, particularly for fibrous adipose tissue; however, surgical outcomes may vary depending on regional anatomical characteristics. **Methods:** This retrospective observational study included five patients with idiopathic posterior neck lipomatosis who underwent UAL using a superwet infiltration technique. Liposuction was performed through three access ports, with initial deep-plane debulking followed by contour refinement. Postoperative outcomes were assessed based on clinical examination, standardized photographic documentation, and patient-reported satisfaction during follow-up. **Results:** The mean follow-up duration was 8.2 months (range, 7–10 months). All patients demonstrated visible improvement in dorsocervical contour following UAL. The mean volume of tumescent infiltration was 176 mL (range, 100–250 mL), and the mean lipoaspirate volume was 178 mL (range, 110–280 mL). Four patients achieved satisfactory contour improvement and reported high satisfaction. One patient exhibited residual dorsocervical prominence consistent with relative undercorrection, despite adequate aspiration volume and ultrasonic application. No major perioperative or postoperative complications were observed. **Conclusions:** Ultrasound-assisted liposuction appeared to provide favorable short-term contour improvement in this small series of idiopathic posterior neck lipomatosis. However, dense fibrous septation and deep fascial tethering in the dorsocervical region may limit complete correction in selected patients, even when aspiration volume and ultrasonic application are considered adequate. Recognition of these anatomical constraints may help refine surgical planning, improve preoperative counseling, and guide expectations regarding residual fullness.

## 1. Introduction

Posterior neck lipomatosis, commonly referred to as a “buffalo hump,” is characterized by abnormal accumulation of adipose and fibrofatty tissue in the dorsocervical region. This condition has been described in a variety of clinical settings, including HIV-associated lipodystrophy, endocrine disorders such as Cushing syndrome, and idiopathic presentations, and may result in both aesthetic deformity and functional discomfort [1,2,3]. Because of its visibility and potential impact on posture, neck mobility, and quality of life, surgical correction of posterior neck lipomatosis remains an important clinical concern.

Several surgical approaches have been proposed for the management of dorsocervical fat pad enlargement, ranging from open excisional lipectomy to suction-assisted liposuction (SAL) and ultrasound-assisted liposuction (UAL). Open excision allows direct removal of fibrofatty tissue and may be effective in selected cases; however, it is associated with longer scars, prolonged recovery, and increased wound-related morbidity [4,5,6]. Consequently, liposuction-based techniques have gained widespread acceptance as less invasive alternatives that can achieve favorable contour improvement with reduced morbidity [7,8,9,10].

Among these techniques, UAL has been increasingly utilized for the treatment of posterior neck lipomatosis, particularly in lesions containing fibrous or dense adipose components. Ultrasonic energy facilitates adipocyte emulsification and may partially disrupt fibrous septa, thereby improving aspiration efficiency compared with conventional liposuction [7,8,9]. Prior studies have reported satisfactory aesthetic outcomes following UAL in appropriately selected patients, supporting its role as a practical and minimally invasive treatment option.

Nevertheless, clinical experience has also demonstrated that surgical outcomes following liposuction can vary, especially in cases with substantial fibrofatty tissue or deep structural involvement [7,8,10]. These observations suggest that, in addition to surgical technique, regional anatomical factors may influence the effectiveness and limitations of liposuction-based approaches.

In this report, we present five cases of idiopathic posterior neck lipomatosis treated with ultrasound-assisted liposuction using a superwet technique. Although most patients achieved favorable contour improvement, one patient demonstrated relative undercorrection despite a substantial aspirate volume and appropriate ultrasonic application. This observation prompted us to examine not merely whether ultrasound-assisted liposuction is feasible, but why correction may remain limited in selected cases. Accordingly, the primary purpose of this study is to highlight dorsocervical anatomical constraints—particularly dense fibrous septation and deep fascial tethering—as clinically relevant limiting factors. By correlating preoperative imaging, physical examination findings, and intraoperative observations, we aim to propose practical preoperative and intraoperative indicators that may help surgeons anticipate residual fullness, refine surgical planning, and improve patient counseling.

## 2. Methods

### 2.1. Study Design and Patients

This retrospective observational study included five patients with idiopathic posterior neck lipomatosis who underwent UAL between October 2022 and May 2025. None of the patients had a history of HIV infection, long-term corticosteroid use, or other systemic conditions known to be associated with dorsocervical lipodystrophy.

The cohort consisted of five women with a mean age of 66.4 years (range, 54–73 years). All patients presented with a visible dorsocervical prominence causing aesthetic concern and, in some cases, discomfort or limitation in neck movement. Preoperative evaluation included detailed medical history, physical examination, and appropriate laboratory or clinical assessment to exclude known secondary causes of dorsocervical fat accumulation, such as endocrine disorders (e.g., Cushing syndrome), HIV-associated lipodystrophy, and long-term corticosteroid use. No clinical or laboratory findings suggestive of secondary causes were identified in this cohort. All lesions were therefore classified as idiopathic posterior neck lipomatosis with suspected fibrofatty components.

In addition, preoperative evaluation included a structured clinical assessment of the dorsocervical lesion, focusing on qualitative features such as compressibility and mobility relative to surrounding tissue. Reduced compressibility or limited mobility was considered suggestive of a more substantial fibrofatty component.

Patient demographic and clinical characteristics are summarized in Table 1.

### 2.2. Surgical Technique

All procedures were performed with the patient in the prone position, with the neck placed in maximal flexion to fully expose the dorsocervical region and facilitate access to the deep fibrofatty tissue. The choice of anesthesia (general versus local anesthesia) was determined based on patient preference and the anticipated operative extent, with procedures performed under general anesthesia in three patients and local anesthesia in two patients. After sterile preparation and draping, three access ports were created at the left shoulder, right shoulder, and caudal midline to allow comprehensive coverage of the posterior neck.

Tumescent infiltration was administered using a superwet technique. The tumescent solution consisted of 1000 mL of Ringer’s lactate solution mixed with 500 mg of lidocaine, 1 mL of epinephrine (1:1000), and 10 mL of 8.4% sodium bicarbonate. A waiting period of approximately 20 min was observed to allow adequate tissue diffusion and vasoconstriction.

Ultrasound-assisted liposuction was performed using an ultrasonic liposuction system (Ultra-Z and ZP-1000, Zerone, Gunpo, Republic of Korea). A 3-mm ultrasound probe was introduced through each access port, and ultrasonic energy was applied sequentially to the targeted dorsocervical tissue in continuous mode. The energy setting was typically adjusted to approximately 80% of the device’s maximum output. The endpoint of ultrasound application was defined as a reduction in resistance within the fibrofatty tissue, as perceived during probe advancement. As a general reference, approximately 1 min of ultrasonic application per 100 mL of tumescent solution was applied; however, the actual duration was adjusted intraoperatively based on tissue characteristics, including the degree of fibrous septation and resistance. Although a general reference for ultrasonic application was used, energy delivery was not strictly standardized and was adjusted according to intraoperative tissue response.

Following ultrasonic emulsification, suction-assisted aspiration was initiated. Initial debulking was performed in the deep plane using a 4-French Mercedes-tip cannula (Zerone, Gunpo, Republic of Korea) to address the deep fibrofatty component. Subsequent refinement was carried out with a 3-French cannula to improve contour smoothness. Superficial liposuction using the 3-French cannula (Zerone, Gunpo, Republic of Korea) was performed selectively and only when necessary, as excessive superficial aspiration was avoided to minimize the risk of contour irregularity. Deep-plane debulking was performed with careful attention to maintaining a consistent plane and avoiding excessive penetration toward deeper structures. Cannula movements were controlled and directed to minimize the risk of injury to adjacent neurovascular structures, particularly in the central dorsocervical region. The intraoperative endpoint was determined based on achieving smooth contour with preservation of adequate superficial tissue thickness.

At the completion of aspiration, the operative field was assessed for symmetry and contour smoothness. Simple dressings were applied to the liposuction port sites. A compression garment was applied and maintained for approximately 4–6 weeks postoperatively. Patients were managed with routine postoperative care and follow-up.

Operative details are summarized in Table 2.

### 2.3. Outcome Assessment

Postoperative outcomes were assessed based on clinical examination, standardized photographic documentation, and patient-reported satisfaction during follow-up visits. Postoperative photographs were obtained in a standardized manner and were reviewed by the operating surgeon. Contour improvement was categorized as satisfactory or undercorrected. Undercorrection was defined as residual dorsocervical prominence identified during follow-up without an initial satisfactory contour improvement, whereas recurrence was defined as reappearance of dorsocervical prominence after an initial satisfactory result. Patient-reported satisfaction was recorded qualitatively during follow-up visits and was not assessed using a formal ordinal or validated questionnaire. Given the retrospective nature of the study and the small cohort size, objective volumetric or scoring systems were not applied.

## 3. Results

### 3.1. Overall Outcomes

The mean follow-up duration was 8.2 months (range, 7–10 months). All five patients demonstrated visible improvement in dorsocervical contour following UAL. The mean volume of tumescent infiltration was 176 mL (range, 100–250 mL), and the mean lipoaspirate volume was 178 mL (range, 110–280 mL).

Four patients reported high satisfaction with the aesthetic outcome and maintained satisfactory contour improvement throughout the follow-up period. One patient (patient No. 4) experienced mild pruritus at the operative site, which persisted for approximately 2 months postoperatively and resolved spontaneously without additional intervention.

One patient (patient No. 3) demonstrated residual dorsocervical prominence consistent with relative undercorrection during follow-up, despite having the highest lipoaspirate volume among the five patients. On preoperative examination, this patient exhibited markedly reduced compressibility and limited mobility of dorsocervical tissue. Despite this finding, the patient reported overall satisfaction with the surgical outcome and did not desire any additional treatment. No major complications, including hematoma, infection, seroma, or neurovascular injury, were observed in any patient.

### 3.2. Representative Case 1

**Figure 1:**
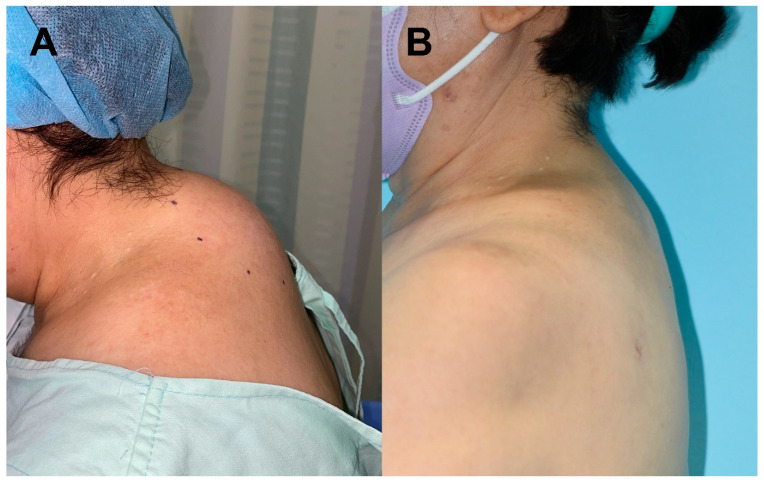
Preoperative and postoperative clinical photographs of dorsocervical lipomatosis. Preoperative photographs were obtained with the neck in a flexed position to better demonstrate the extent of dorsocervical lipomatosis, whereas postoperative photographs were taken in a neutral position to assess contour improvement in a natural posture. (**A**) Preoperative photograph demonstrating a prominent dorsocervical fat pad. (**B**) Postoperative photograph obtained 8 months after ultrasound-assisted liposuction, showing marked improvement in dorsocervical contour and a smooth cervicothoracic profile.

**Figure 2:**
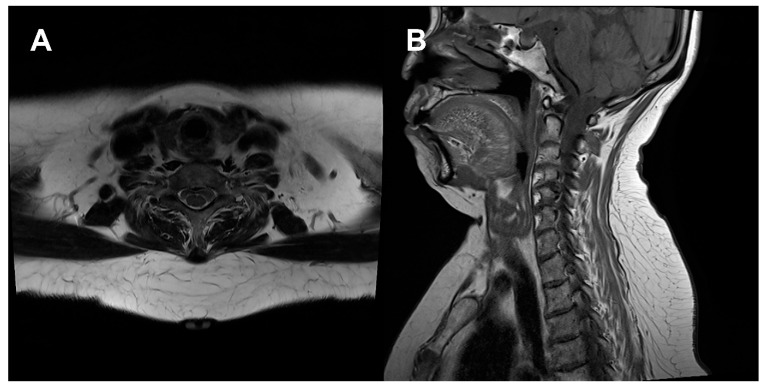
Preoperative T1-weighted magnetic resonance imaging (MRI) of patient 1. (**A**) Axial view demonstrating a dorsocervical fat pad with poorly defined margins relative to the surrounding adipose tissue, without a discrete encapsulated mass. (**B**) Sagittal view showing indistinct boundaries between the superficial and deep adipose layers, with multiple internal linear low-signal strands within the deep portion, consistent with dense fibrous septation.

Patient No. 1 was a 68-year-old woman with no relevant medical comorbidities who presented with idiopathic posterior neck lipomatosis. Preoperative evaluation revealed no secondary causes. Clinical photographs obtained in the lateral view demonstrated a prominent dorsocervical contour deformity (Figure 1A). Preoperative magnetic resonance imaging (MRI) revealed a well-developed dorsocervical fat pad with fibrofatty components extending into the deep layer, as demonstrated on axial and sagittal T1-weighted images (Figure 2). Based on these findings, UAL was planned.

The procedure was performed under general anesthesia using a superwet technique. A total of 150 mL of tumescent solution was infiltrated, followed by UAL, yielding 120 mL of lipoaspirate. The intraoperative course was uneventful.

At 8 months postoperatively, lateral-view clinical photographs demonstrated marked improvement in dorsocervical contour with restoration of a smooth cervicothoracic transition (Figure 1B). No postoperative complications were observed, and the patient reported high satisfaction with the aesthetic outcome.

### 3.3. Representative Case 2

**Figure 3:**
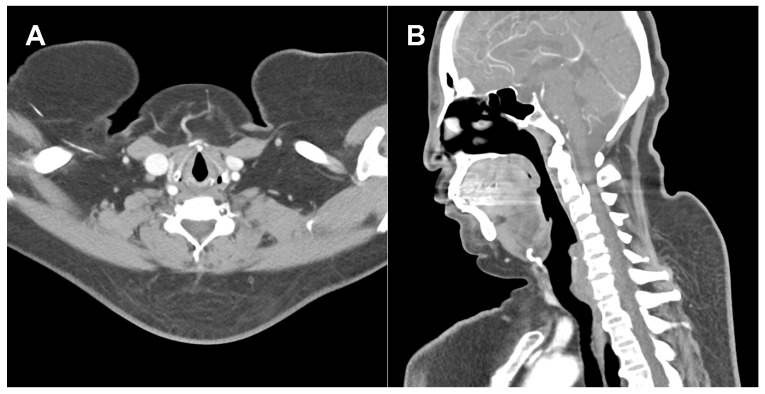
Preoperative computed tomography (CT) of patient 3. (**A**) Axial view demonstrating a prominent dorsocervical fat pad with heterogeneous internal architecture and multiple linear soft-tissue strands. (**B**) Sagittal view showing posterior neck fat accumulation with conspicuous internal septation within the deep portion, findings suggestive of well-developed fibrous septa. Compared with Representative Case 1, the fibrous components appear more conspicuous, which may correlate with the relative resistance encountered intraoperatively and the subsequent undercorrected outcome.

Patient No. 3 was a 65-year-old woman with a medical history of hypertension and diabetes mellitus who presented with idiopathic posterior neck lipomatosis. Preoperative evaluation did not reveal any secondary causes. On preoperative examination, the lesion was firm, poorly compressible, and demonstrated limited mobility relative to the surrounding tissue. Preoperative computed tomography (CT) imaging demonstrated a prominent dorsocervical fat pad (Figure 3).

The procedure was performed under general anesthesia using a superwet technique. A total of 230 mL of tumescent solution was infiltrated, followed by ultrasound-assisted liposuction, yielding 280 mL of lipoaspirate. Intraoperatively, the dorsocervical tissue was noted to be firm, deeply seated, and relatively resistant to cannula passage, particularly in the central region.

During follow-up, the patient demonstrated residual dorsocervical prominence consistent with relative undercorrection. Despite this finding, the patient reported overall satisfaction with the aesthetic outcome and did not desire any additional treatment. No procedure-related complications were observed.

## 4. Discussion

Ultrasound-assisted liposuction has been widely adopted as an effective and minimally invasive technique for the management of posterior neck lipomatosis, particularly in patients with dense or fibrous adipose tissue. Previous clinical series have demonstrated that ultrasonic energy facilitates adipocyte emulsification and partial disruption of fibrous septa, thereby improving contour outcomes compared with conventional suction-assisted liposuction while avoiding the morbidity associated with open excisional approaches [7,8,9]. Consistent with these reports, the majority of patients in the present series achieved satisfactory contour improvement and high postoperative satisfaction following UAL combined with a superwet infiltration technique.

Despite these favorable outcomes, one patient in our series demonstrated relative undercorrection. Importantly, this residual dorsocervical fullness was not attributable to insufficient aspiration volume or inadequate application of ultrasonic energy. Notably, the undercorrected case had the highest lipoaspirate volume, suggesting that insufficient aspiration was unlikely to be the primary cause. Instead, intraoperative findings indicated that the remaining tissue was deeply seated, firm, and poorly mobile, particularly in the central dorsocervical region [3,8,10]. These findings suggest that regional structural characteristics may limit the effectiveness of liposuction, even when advanced energy-assisted techniques are employed.

In this context, preoperative physical examination may provide clinically relevant insight into underlying tissue characteristics. Reduced compressibility and limited mobility may serve as practical indicators of dense fibrous septation and deep fascial tethering, which can limit the effectiveness of liposuction-based techniques.

The dorsocervical region exhibits unique anatomical features that distinguish it from more compliant subcutaneous fat compartments. Anatomical studies have shown that adipose tissue in the posterior neck is interlaced with thick, vertically oriented fibrous septa and firmly anchored to the underlying deep cervical fascia and nuchal ligament [3,11]. This structural framework compartmentalizes adipose tissue and restricts its mobility, particularly within the central and deep layers. In lipomatosis, these characteristics may be further accentuated by increased fibrous content and ill-defined boundaries between pathological and adjacent normal fat. Such architecture can limit cannula mobility and reduce the efficiency of fat emulsification and aspiration in deeper planes [12]. These observations should be interpreted as a clinical–anatomical correlation rather than a direct mechanistic demonstration.

From a practical standpoint, several imaging features may help anticipate technical difficulty and the likelihood of residual fullness. These include increased depth of the dorsocervical fat pad, prominent internal fibrous septation, indistinct boundaries between superficial and deep adipose layers, and imaging findings suggestive of adherence to the underlying deep fascia. Lesions demonstrating these features may be associated with increased intraoperative resistance and a higher likelihood of incomplete correction with liposuction-based techniques.

In addition to conventional imaging modalities, dermoscopy-guided high-frequency ultrasound (20–40 MHz) has been introduced as a novel imaging modality capable of visualizing superficial soft tissue structures, including fibrous components. Although primarily used in dermatologic and oncologic settings, it may have potential utility in the preoperative assessment of fibrofatty lesions. In particular, it may facilitate visualization of fibrous septation that could contribute to intraoperative resistance. Further studies are warranted to determine its practical role in surgical planning for dorsocervical lipomatosis [13,14].

This undercorrected case may reflect regional structural resistance rather than technical failure of UAL. Although UAL enhances the surgeon’s ability to address fibrous adipose tissue compared with conventional liposuction, it does not completely overcome the constraints imposed by deep fascial tethering. Aggressive pursuit of complete removal under these conditions may require deeper dissection or excessive force, thereby increasing the risk of contour irregularity or injury to adjacent structures. Accordingly, achieving an appropriate balance between maximal volume reduction and procedural safety remains a critical consideration.

This experience offers practical guidance for both preoperative assessment and intraoperative decision-making. On physical examination, lesions demonstrating good compressibility and mobility may be more amenable to liposuction-based treatment alone. In contrast, lesions with reduced compressibility or limited mobility may indicate a substantial deep component and potential resistance to liposuction. When such features are present, surgeons should anticipate technical limitations and counsel patients regarding the possibility of residual fullness. In selected cases, a more deliberate deep-plane strategy, involving targeted ultrasonic energy delivery to disrupt fibrous septation followed by controlled mechanical debulking of the deep fibrofatty tissue using suction-assisted aspiration, may be considered to optimize outcomes [10,12,15,16].

Notably, the patient with relative undercorrection in the present series remained satisfied with the aesthetic result and declined additional intervention, underscoring that complete volumetric elimination may not be necessary to achieve meaningful clinical and patient-reported improvement. This observation highlights the importance of individualized treatment goals and realistic expectation-setting in the management of posterior neck lipomatosis.

This study has several limitations. First, the small sample size and retrospective design limit the generalizability of the findings. Second, outcome assessment was based on clinical examination, photographic evaluation, and patient-reported satisfaction, without the use of objective or validated quantitative measures, which represents an inherent limitation of the study. Third, lipoaspirate volume was not normalized to patient-specific factors such as body mass index, lesion size, or imaging-estimated fat volume, which may influence the interpretation of volume–outcome relationships. Fourth, the relatively short follow-up period makes it difficult to distinguish persistent undercorrection from potential late recurrence, as longer-term changes may be influenced by ongoing fat accumulation or underlying etiologic factors. Finally, all patients were older women with idiopathic posterior neck lipomatosis, which may limit generalizability to other etiologic subgroups. In addition, differences in head positioning between preoperative and postoperative photographs may limit direct visual comparison. Preoperative images were intentionally obtained with neck flexion to better demonstrate lesion extent, whereas postoperative images were taken in a neutral position to assess contour in a functional posture. Despite these limitations, the present study emphasizes the integration of preoperative clinical assessment, imaging findings, and intraoperative observations to provide practical indicators for anticipating anatomical limitations.

## 5. Conclusions

In summary, ultrasound-assisted liposuction appears to be a feasible and minimally invasive treatment option that can provide favorable contour improvement for posterior neck lipomatosis, particularly in cases involving fibrous adipose tissue. However, dense fibrous septation and deep fascial tethering in the dorsocervical region can influence surgical outcomes in select patients. Recognition of these anatomical constraints allows surgeons to refine operative planning, optimize patient counseling, and achieve safer and more predictable results.

## Figures and Tables

**Table 1:** Patient demographic and clinical characteristics.

Patient No.	Age (Years)	Sex	BMI (kg/m^2^)	Comorbidities	Preoperative Evaluation	Follow-Up Duration (Months)
1	68	F	28.8	None	MRI	8
2	72	F	28.7	HBP	MRI	8
3	65	F	30.9	HBP, DM	CT	8
4	54	F	33.1	HBP	MRI	10
5	73	F	29.7	HBP	MRI	7

Abbreviations: BMI = body mass index; F = female; HBP = high blood pressure; DM = diabetes mellitus; MRI = magnetic resonance imaging; CT = computed tomography.

**Table 2:** Operative details and surgical outcomes of ultrasound-assisted liposuction.

Patient No.	Anesthesia Type	Tumescent Infiltration Volume (mL)	Lipoaspirate Volume (mL)	Contour Outcomes	Complications
1	General	150	120	Satisfactory	None
2	Local	250	200	Satisfactory	None
3	General	230	280	Undercorrected	None
4	General	150	180	Satisfactory	Mild pruritus
5	Local	100	110	Satisfactory	None

## Data Availability

The data presented in this study are available on reasonable request from the corresponding author. The data are not publicly available due to privacy restrictions related to patient information.
